# Comparing therapeutic effects across tyrosine kinase inhibitors: Chronic myeloid leukemia outcomes and analysis of influencing factors

**DOI:** 10.1097/MD.0000000000043120

**Published:** 2025-07-18

**Authors:** Zizhong Zhang, Weiwei Jiang, Sen Ding, Xuliang Shen

**Affiliations:** a Department of Haematology, Heping Hospital Affiliated to Changzhi Medical College, Shanxi Clinical Medical Research Center for Hematologic Diseases (Myeloproliferative Neoplasms), Changzhi, China; b Jining Institute of Education, Jining, China.

**Keywords:** chronic myeloid leukaemia, efficacy, flumatinib, imatinib, influencing factors, nilotinib

## Abstract

This study aims to comprehensively assess the effects of imatinib, nilotinib, and flumatinib in treating chronic myeloid leukaemia and to explore the main factors affecting its efficacy. Ninety-nine chronic myeloid leukaemia patients initially diagnosed and treated with one of these 3 tyrosine kinase inhibitors at a tertiary hospital in Shanxi Province between June 2018 and June 2023 were selected and divided into an imatinib group (n = 32), nilotinib group (n = 30), and flumatinib group (n = 37). Hematological response rates, cytogentic response rates, molecular response rates, and adverse reactions were compared among the 3 groups to statistically analyze efficacy and safety. Univariate analysis and logistic regression were used to explore the related factors influencing the curative effect. A nomogram prediction model of influencing factors of efficacy was constructed in R software and validated according to receiver operating characteristic and calibration curves, with a clinical decision curve and clinical impact curve further drawn to confirm its clinical practicability. The complete cytogenetic response at 3 months differed significantly, with rates of 53.13%, 76.67%, and 78.38% for the imatinib, nilotinib, and flumatinib groups, respectively (*P* < .05). Major molecular response (MMR) rates at 3 months were 25.00%, 53.33%, and 51.35%, reaching 78.13%, 90.00%, and 83.78% at 12 months, respectively. Deep molecular response (DMR) rates at 12 months were 50.00%, 76.67%, and 75.68% in each respective group (*P* < .05). Multivariate logistic regression indicated early molecular response, white blood cell count, red cell distribution width and platelet count as independent influencing factors of MMR. Age, drug type, early early molecular response, and red cell distribution width were identified as independent influencing factors of DMR (*P* < .05). The areas under the receiver operating characteristic curves for MMR and DMR nomogram models were 0.912(95% confidence interval: 0.833, 0.990)and 0.874 (95% confidence interval: 0.801, 0.946), respectively, indicating satisfactory model calibration. Nilotinib and flumatinib demonstrate superior efficacy over imatinib, with effectiveness influenced by various factors including sociodemographic characteristics, clinical heterogeneity, and drug side effects. The proposed clinical prediction model may provide valuable insights for decision-making and demonstrates generalizability and practical application value.

## 1. Introduction

Chronic myeloid leukemia (CML) is a malignancy of hematopoietic stem cells driven by BCR-ABL fusion gene abnormality. This results from a t(9;22)(q34;q11.2) chromosomal translocation mechanism, which disrupts normal cell regulation by abnormally activating key signaling pathways such as PI3K, MAPK, and JAK/STAT, leading to uncontrolled cell proliferation and apoptosis dysfunction. This pathological process not only results in abnormal accumulation of granulocytes in the bone marrow, but also severely compromises normal hematopoietic function, posing a significant threat to patients’ lives.^[1,2]^

Tyrosine kinase inhibitors (TKI) such as imatinib and nilotinib are essential in treating CML. These therapies precisely target tyrosine kinase activity in malignant cells, effectively suppressing their proliferation, greatly improving patients’ quality of life, and extending survival.^[3]^ However, the response to TKI therapy varies greatly among patients in clinical settings; some maintain long-term stability while others experience disease progression, drug resistance, and relapse. This variability underscores the complex nature of treatment efficacy, introducing challenges and uncertainties in clinical decision-making.^[4]^

This study focuses on patients in the chronic disease phase of newly diagnosed CML, aiming to systematically analyze the factors influencing the efficacy of TKI therapy. By conducting a comprehensive review and analysis of case data from a local center, this study seeks to uncover the underlying mechanisms of differences in treatment efficacy. The goal is to secure a robust theoretical foundation and practical guidance for individualized treatment strategies in CML, assist clinical practitioners in optimizing therapeutic approaches, and ultimately improve prognoses.

## 2. Materials and methods

### 2.1. Subjects

A retrospective analysis was conducted on 99 patients diagnosed with chronic-phase CML (CML-CP) at a tertiary hospital in Shanxi Province from June 2018 to June 2023, all of whom received treatment with imatinib, nilotinib, or flumatinib as initial therapy.

Inclusion criteria were: (1) patients meeting WHO diagnostic criteria for CML-CP^[5]^; (2) having initiated treatment with imatinib, nilotinib, or flumatinib; (3) age ≥ 18 years; (4) good treatment compliance, normal cognitive function, and ability to complete relevant examinations; (5) patients and their families having been informed about the study and providing written consent.

Exclusion criteria included: (1) prior treatment with non-tyrosine kinase inhibitors; (2) patients progressing to accelerated phase/blast crisis or mortality during follow-up; (3) patients lost to follow-up; (4) inability to cooperate with data collection due to personal reasons (e.g., mental illness, communication difficulties); (5) severe heart, liver, lung, kidney, or other diseases impacting daily life.

This study was approved by the Ethics Committee of our hospital, the approval number is: (2024)082.

### 2.2. Treatment options

Imatinib group: imatinib 400 mg once daily; nilotinib group: nilotinib 300 mg twice daily; flumatinib group: flumatinib 600 mg once daily. During the treatment, the dose was adjusted or discontinued according to individual tolerance and blood test indicators.

### 2.3. Evaluation indicators

Efficacy was assessed according to the 2013 and 2020 European Leukemia Network (ELN) guidelines.^[6,7]^ Treatment was considered effective if the patient exhibited the following responses.

1. Complete hematologic response: This involves a peripheral white blood cell count below 10 × 10^9^/L, platelet count below 450 × 10^9^/L, no myeloid immature cells present in peripheral blood, basophils <5%, no symptoms or signs of disease, and resolved palpable splenomegaly.
2. Cytogenetic response: A complete cytogenetic response (CCyR) includes no Ph + metaphases and a major cytogenic response involves 0% to 35% of the Ph + chromosomes in metaphase (fission phase).
3. Molecular response: Early molecular remission (EMR) at 3 and 6 months with BCR-ABLIS ≤ 10%; major molecular remission (MMR) at 3, 6, 9, and 12 months with BCR-ABLIS ≤ 0.1%; deep molecular remission (DMR) at 9 and 12 months with BCR-ABLIS ≤ 0.01%.

### 2.4. Quality control

The following measures were taken to ensure data quality as well as patients’ safety:

1. Patients provided informed consent according to the principles of medical ethics.
2. Patients were enrolled according to the stated inclusion and exclusion criteria.
3. Data were collected between June 2018 and June 2024, encompassing inpatient and outpatient personal information and test results, with follow-ups aftert least 1 year.
4. Double-entry and double-check methods were used to ensure data was entered accurately.

### 2.5. Statistical methods

Statistical analyses were conducted in SPSS 26.0 software. Measurement data were confirmed to follow a normal distribution and are expressed here as ($\bar{x}$±S). An independent samples *t* test was conducted for comparisons between 2 groups, and the analysis of variance was used for comparisons among multiple groups. Count data are expressed as n (%). Between-group comparisons were performed using a chi-square test of Fisher exact test as appropriate. Factors influencing the curative effect were screened through single factor analysis, then incorporated into the logistic regression model for multi-factor analysis. A nomogram prediction model of influencing factors was constructed in R software and validated according to receiver operating characteristic (ROC) and calibration curves, with a clinical decision curve (DCA) and clinical impact curve further drawn to confirm its clinical practicability.

## 3. Result

### 3.1. General clinical data

A total of 99 CML patients were enrolled, including 32 patients in imatinib group, 30 patients in nilotinib group and 37 patients in flumatinib group (Table 1). There were no significant difference in general data among the 3 groups *(P* < .05).

**Table 1:** General data of CML patients [$\bar{x}$±S/n(%)].

Item	Imatinib group(n = 32)	Nilotinib group(n = 30)	Flumatinib group(n = 37)	*X*^2^/*H*/*F*	*P*
Sex [n (%)]				1.653	.466
Male	13 (40.63)	17 (56.67)	17 (45.95)		
Female	19 (59.37)	13 (43.33)	20 (54.05)		
Age ($\bar{x}$ ± *s*, years)	51.50 ± 14.73	47.07 ± 11.80	50.27 ± 13.63	0.891	.414
Height ($\bar{x}$ ± *s*, cm)	163.91 ± 7.00	166.33 ± 7.74	166.35 ± 9.33	0.966	.384
Weight ($\bar{x}$ ± *s*, kg)	63.47 ± 9.23	66.47 ± 13.77	63.43 ± 10.71	0.745	.478
BMI ($\bar{x}$ ± *s*, kg/m^2^)	23.63 ± 3.10	24.01 ± 4.72	22.80 ± 2.32	1.095	.339
Academic [n(%)]				4.841	.312
Middle school and below	16 (50.00)	7 (23.33)	15 (40.54)		
High school	8 (25.00)	12 (40.00)	12 (32.43)		
College and above	8 (25.00)	11(36.67)	10(31.25)		
Smoking [n(%)]	3 (9.38)	4 (13.33)	5 (13.51)	0.415*	.861
Alcohol [n(%)]	3 (9.38)	3 (10.00)	6 (16.22)	0.886*	.738
Blood type [n(%)]				2.598	.864
A	8 (25.00)	6 (20.00)	9 (24.32)		
B	7 (21.88)	7 (23.33)	12 (32.43)		
AB	9 (28.12)	7 (23.33)	6 (16.22)		
O	8 (25.00)	10 (33.34)	10 (27.03)		
Splenomegaly [n(%)]				9.276*	.154
No	16 (50.00)	10 (33.33)	11 (29.73)		
Mild	1 (3.13)	3 (10.00)	9 (24.32)		
Moderate	8 (25.00)	6 (20.00)	7 (18.92)		
Severe	7 (21.87)	11 (36.67)	10 (27.03)		
Complication [n(%)]					
Hypertensive	7 (21.87)	7 (23.33)	10 (27.03)	0.267	.876
Diabetes	1 (3.13)	1 (3.33)	3 (8.11)	1.038*	.620
Heart disease	3 (9.38)	1 (3.33)	3 (8.11)	0.997*	.701
Sokal score [n(%)]				4.128*	.398
Low risk	15 (46.88)	15 (50.00)	17 (45.94)		
Moderate risk	14 (43.75)	14 (46.67)	13 (35.14)		
High risk	5 (15.63)	1 (3.33)	7 (18.92)		
ELTS score [n(%)]				2.800*	.613
Low risk	23 (71.88)	17 (56.67)	20 (54.05)		
Moderate risk	8 (25.00)	11 (36.67)	14 (37.84)		
High risk	1 (3.12)	2 (6.66)	3 (8.11)		
EURO score [n(%)]				9.019*	.052
Low risk	13 (40.62)	10 (33.33)	19 (51.35)		
Moderate risk	15 (46.88)	20 (66.67)	13 (35.14)		
High risk	4 (12.50)	0 (0.00)	5 (13.51)		
EUTOS score [n(%)]				0.236*	.940
Low risk	27 (84.38)	25 (83.33)	32 (86.49)		
High risk	5 (15.62)	5 (16.67)	5 (13.51)		

BMI = body mass index.

* Fisher exact test.

### 3.2. Efficacy results

Hematological and cytogenetic responses, as well as BCR-ABL1 quantitative results, were collected for 99 patients. At 12 months, all patients had achieved complete hematologic response. A total of 81 patients (81.82%) achieved major cytogenic response and 69 (69.70%) achieved CCyR. Additionally, all patients reached EMR, with 83 patients (83.84%) reaching MMR and 67 (67.68%) reaching DMR.

#### 3.2.1. Complete hematologic response

At 3 months,the complete hematologic response rates for imatinib, nilotinib, and flumatinib groups were 19 patients (59.38%), 20 patients (66.67%), 26 patients (70.27%), respectively, and 31 cases (96.88%), 30 cases (100%), 37 cases (100%) at 12 months of treatment, respectively, and the difference was not statistically significant (*P* > .05).

#### 3.2.2. Cytogenetic response

At 3 months, the major cytogenic response rates for imatinib, nilotinib, and flumatinib groups were 22 patients (68.75%), 26 patients (86.67%), and 33 patients (89.19%), respectively, with no statistically significant difference among them (*P* < .05). CCyR rates were 17 patients (53.13%), 23 patients (76.67%), and 29 patients (78.38%), respectively, showing a statistically significant difference (*P *< .05).

#### 3.2.3. Molecular response

The MMR rates at 3 months were 25.00% (n = 8), 53.33% (n = 16), and 51.35% (n = 19), reaching 78.13% (n = 25), 90.00% (n = 27), and 83.78% (n = 31) at 12 months, respectively. DMR rates at 12 months were 50.00% (n = 16), 76.67% (n = 23), and 75.68% (n = 28) in each respective group (*P* < .05). Details are provided in Table 2.

**Table 2:** Treatment response in CML patients [n(%)].

Item	Months	Imatinib group(n = 32)	Nilotinib group(n = 30)	Flumatinib group(n = 37)	*χ* ^*2*^	*P*
Hematologic response						
CHR	3	19 (59.38)	20 (66.67)	26 (70.27)	0.923	.646
	6	24 (75.00)	26 (86.67)	33 (89.19)	2.804	.311
	9	30 (93.75)	29 (96.67)	35 (94.59)	0.469*	.865
	12	31 (96.88)	30 (100.00)	37 (100.00)	1.911*	.626
Cytogenetic response						
MCyR	3	22 (68.75)	26 (86.67)	33 (89.19)	5.499	.063
CCyR	3	17 (53.13)	23 (76.67)	29 (78.38)	6.171	.046
Molecular response						
EMR	3	24 (75.00)	29 (96.67)	33 (89.19)	6.651*	.036
	6	31 (96.88)	30 (100.00)	35 (94.59)	1.488*	.774
MMR	3	8 (25.00)	16 (53.33)	19 (51.35)	6.567	.041
	6	14 (43.75)	21 (70.00)	26 (70.27)	6.382	.042
	9	21 (65.63)	25 (83.33)	28 (75.68)	2.599	.294
	12	25 (78.13)	27 (90.00)	31 (83.78)	1.612	.491
DMR	9	14 (43.75)	17 (56.67)	17 (45.95)	1.187	.628
	12	16 (50.00)	23 (76.67)	28 (75.68)	6.761	.037

CCyR = complete cytogenetic response, CHR = complete hematologic response, DMR = deep molecular response, EMR = early molecular response, MCyR = major cytogenetic response, MMR = major molecular response.

* Fisher exact test.

### 3.3. Adverse drug reactions

Most adverse reactions within the 3 groups were mild to moderate (grade 1–2), with higher overall incidences in the imatinib group compared to the other 2 groups. Among hematologic adverse events, anemia and leukopenia were more frequent in the imatinib group, while anemia and thrombocytopenia were more common in nilotinib and flumatinib groups. Pruritus was a frequent non-hematologic adverse reaction across all 3 groups. Edema was more prevalent in the imatinib group, liver dysfunction was more common in the nilotinib group, and diarrhea occurred most frequently in the flumatinib group. One case of pleural effusion (grade 1–2) occurred in the imatinib group and nilotinib group each. Most adverse reactions were manageable and improved shortly after dose reduction or temporary treatment withdrawal. Details are provided in Table 3.

**Table 3:** Adverse reactions in the 3 groups [n(%)].

Adverse reactions	Imatinib group (n = 32)		Nilotinib group (n = 30)		Flumatinib group(n = 37)	
	Grade 1–2	Grade 3–4	Grade 1–2	Grade 3–4	Grade 1–2	Grade 3–4
Hematologic adverse events						
Hypoleucocytosis	8 (25.00)	1 (3.13)	3 (10.0)	1 (3.33)	5 (13.51)	0 (0.00)
Thrombopenia	5 (15.63)	0 (0.00)	6 (20.0)	4 (13.33)	6 (16.22)	1 (2.70)
Anemia	10 (31.25)	1 (3.13)	7 (23.33)	2 (6.67)	4 (10.81)	2 (5.41)
Non-hematologic adverse events						
Liver dysfunction	5 (15.63)	0 (0.00)	14 (46.67)	0 (0.00)	6 (16.22)	0 (0.00)
Renal dysfunction	1 (3.13)	0 (0.00)	1 (3.33)	0 (0.00)	3 (8.11)	0 (0.00)
ECG abnormality	2 (6.25)	0 (0.00)	1 (3.33)	0 (0.00)	1 (2.70)	0 (0.00)
Edema	17 (53.13)	1 (3.13)	4 (13.33)	0 (0.00)	3 (8.11)	0 (0.00)
Nausea and vomit	12 (37.50)	1 (3.13)	5 (16.67)	0 (0.00)	4 (10.81)	0 (0.00)
Encephalalgia	1 (3.13)	0 (0.00)	6 (18.18)	0 (0.00)	4 (10.81)	0 (0.00)
Musculoskeletal-pain	8 (25.00)	1 (3.13)	11 (36.67)	0 (0.00)	6 (16.22)	0 (0.00)
Rash	9 (28.13)	1 (3.13)	12 (40.00)	2 (6.67)	8 (21.62)	1 (2.70)
Diarrhea	7 (21.88)	1 (3.13)	0 (0.00)	0 (0.00)	12 (32.43)	1 (2.70)
Fatigue	9 (28.13)	1 (3.13)	9 (30.00)	0 (0.00)	8 (21.62)	0 (0.00)
Pleural effusion	1 (3.13)	0 (0.00)	1 (3.33)	0 (0.00)	0 (0.00)	0 (0.00)

ECG = electrocardiogram.

### 3.4. Influencing factors of efficacy

#### 3.4.1. Influencing factors

According to the recommendations of the 2013 and 2020 ELN guidelines,^[6,7]^ the molecular response was the most important indicator to evaluate the treatment response of patients. Univariate analysis indicated that age, early EMR, white blood cell count (WBC), platelet count (PLT), and red cell distribution width (RDW) at diagnosis influenced 12-month MMR. Additional factors affecting 12-month DMR included age, educational background, splenomegaly, drug type, early EMR, white blood cell count, hemoglobin, RDW, and lactate dehydrogenase at initial diagnosis (*P* < .05). Details are provided in Table 4.

**Table 4:** Univariate analysis of the effect of treatment [n(%)].

Influencing factors	12-month MMR				12-month DMR			
	Yes	No	*χ*^*2*^/*t*	*P*	Yes	No	*χ*^*2*^/*t*	*P*
Age ($\bar{x}$ ± *s*, years)			4.970	.035			12.313	.001
≥60	19 (22.89)	8 (50.00)			11 (16.42)	16 (50.00)		
<60	64 (77.11)	8 (50.00)			56 (83.58)	16 (50.00)		
Academic [n(%)]			1.670	.457			6.417	.046
Middle school and below	31 (37.35)	7 (43.75)			20 (29.85)	18 (56.24)		
High school	29 (34.94)	3 (18.75)			25 (37.31)	7 (21.88)		
College and above	23 (27.71)	6 (37.50)			22 (32.83)	7 (21.88)		
Splenomegaly [n(%)]			2.319*	.529			8.701	.032
No	32 (38.56)	5 (31.25)			28 (41.79)	9 (28.12)		
Mild	11 (13.25)	2 (22.50)			11 (16.42)	2 (6.25)		
Moderate	19 (22.89)	2 (22.50)			15 (22.39)	6 (18.75)		
Severe	21 (25.30)	7 (43.75)			13 (19.40)	15 (46.88)		
Drug types			1.139	.382			6.754	.012
First generation TKI	25 (30.12)	7 (43.75)			16 (23.88)	16 (50.00)		
Second generation TKI	58 (69.88)	9 (56.25)			51 (76.12)	16 (50.00)		
Early EMR			26.760†	.001			16.056†	.001
Yes	79 (95.18)	7 (43.75)			65 (97.02)	21 (65.62)		
No	4 (4.82)	9 (56.25)			2 (2.98)	11 (34.38)		
WBC(10^9^/L)			7.298†	.007			4.269	.039
100	29 (34.94)	12 (25.00)			25 (37.31)	19 (59.38)		
<100	54 (65.06)	4 (75.00)			42 (62.69)	13 (40.62)		
HGB(g/L)			3.497	.061			6.287	.012
≥90	52 (62.65)	6 (37.50)			45 (67.16)	13 (40.62)		
<90	31 (37.35)	10 (62.50)			22 (32.84)	19 (59.38)		
PLT(10^9^/L)			4.062	.044			0.209	.648
≥450	25 (30.12)	9 (56.25)			22 (32.84)	12 (37.25)		
<450	58 (69.88)	7 (43.75)			45 (67.16)	20 (62.65)		
RDW(%)			4.566	.033			6.243	.012
≥18	33 (39.76)	11 (68.75)			24 (35.82)	20 (62.50)		
<18	50 (60.24)	5 (31.25)			43 (64.18)	12 (37.50)		
LDH(IU/L)			3.497	.061			6.287	.012
≥1000	31 (37.35)	10 (62.50)			22 (32.84)	19 (59.38)		
<1000	52 (62.65)	6 (37.50)			45 (67.16)	13 (40.62)		

DMR = deep molecular response, HGB = hemoglobin, LDH = lactate dehydrogenase, PLT = platelet, RDW = red cell distribution width, WBC = white blood cell count.

* Fisher exact test.

† Correction of continuity.

According to the results of univariate analysis, meaningful indicators were incorporated into the binary logistic regression model. Multivariate logistic regression indicated early EMR, WBC, RDW, and PLT as independent influencing factors of MMR (Table 5). Age, drug type, early EMR, and RDW were identified as independent influencing factors of DMR (*P* < .05) (Table 6).

**Table 5:** The multivariate logistic regression model of MMR.

Item	*β*	SE	*Wald*	*P*	*OR*	95% CI	
						Lower limit	Upper limit
Age	‐1.573	0.881	3.187	0.074	0.207	0.037	1.167
Early EMR	3.814	1.015	14.131	0.001	45.336	6.206	331.201
WBC	‐2.037	0.875	5.420	0.020	0.130	0.023	0.725
RDW	‐1.886	0.944	3.988	0.046	0.152	0.024	0.966
PLT	‐2.167	0.853	6.457	0.011	0.115	0.022	0.609
Constant	2.672	1.233	4.694	0.030	14.465		

95% CI = 95% confidence interval, OR = odds ratio, PLT = platelet, RDW = red cell distribution width, SE = standard error, WBC = white blood cell count, β = beta.

**Table 6:** The multivariate logistic regression model of DMR.

Item	*β*	SE	*Wald*	*P*	*OR*	*95% CI*	
						Lower limit	Upper limit
Age	-1.984	0.717	7.648	.006	0.138	0.034	0.561
Academic (middle school and below as reference)			0.725	.696			
High school	‐0.635	0.798	0.633	.426	0.530	0.111	2.533
College and above	‐0.586	0.839	0.488	.485	0.556	0.107	2.882
Splenomegaly (Asplenomegaly as reference)			3.513	.319			
Mild	1.434	0.983	2.131	.144	4.197	0.612	28.791
Moderate	1.628	1.183	1.892	.169	5.093	0.501	51.793
Severe	1.108	0.826	1.801	.180	3.028	0.600	15.272
Drug types	1.529	0.710	4.639	.031	4.614	1.148	18.551
Early EMR	2.528	1.016	6.191	.013	12.531	1.710	91.815
WBC	-0.854	0.781	1.196	.274	0.426	0.092	1.967
HGB	0.278	0.761	0.133	.715	1.320	0.297	5.863
RDW	‐1.403	0.691	4.116	.042	0.246	0.063	0.953
LDH	0.100	0.831	0.014	.904	1.105	0.217	5.633
Constant	‐2.791	1.842	2.297	.130	0.061		

95% CI = 95% confidence interval, DMR = deep molecular response, HGB = hemoglobin, LDH = lactate dehydrogenase, OR = odds ratio, RDW = red cell distribution width, SE = standard error, WBC = white blood cell count, β = beta.

#### 3.4.2. Nomogram prediction model

**Figure 1.**
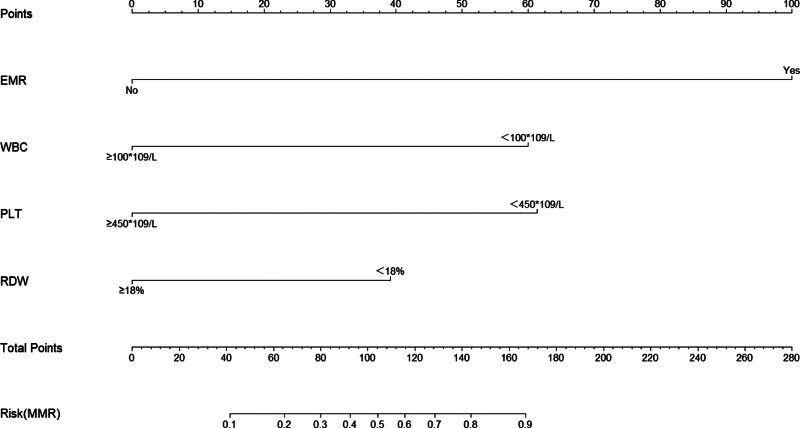
Nomogram prediction model of MMR. MMR = major molecular response.

**Figure 2.**
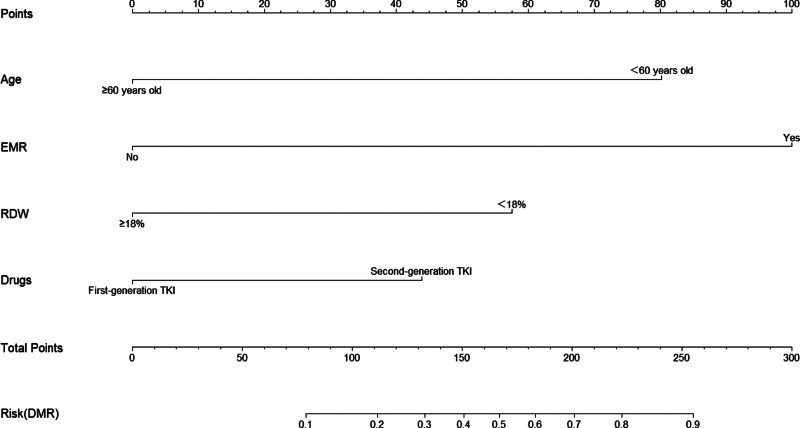
Nomogram prediction model of DMR. DMR = deep molecular response.

The identified influencing factors for MMR (Fig. 1) and DMR (Fig. 2) were incorporated into a nomogram prediction model. Each factor’s specific value was used to obtain a corresponding score on the “points” scale. These scores were summed to derive a “total points score,” placed on the “total points scale,” to determine the predicted probability of the corresponding outcome event.

#### 3.4.3. Model fit assessments

**Figure 3.**
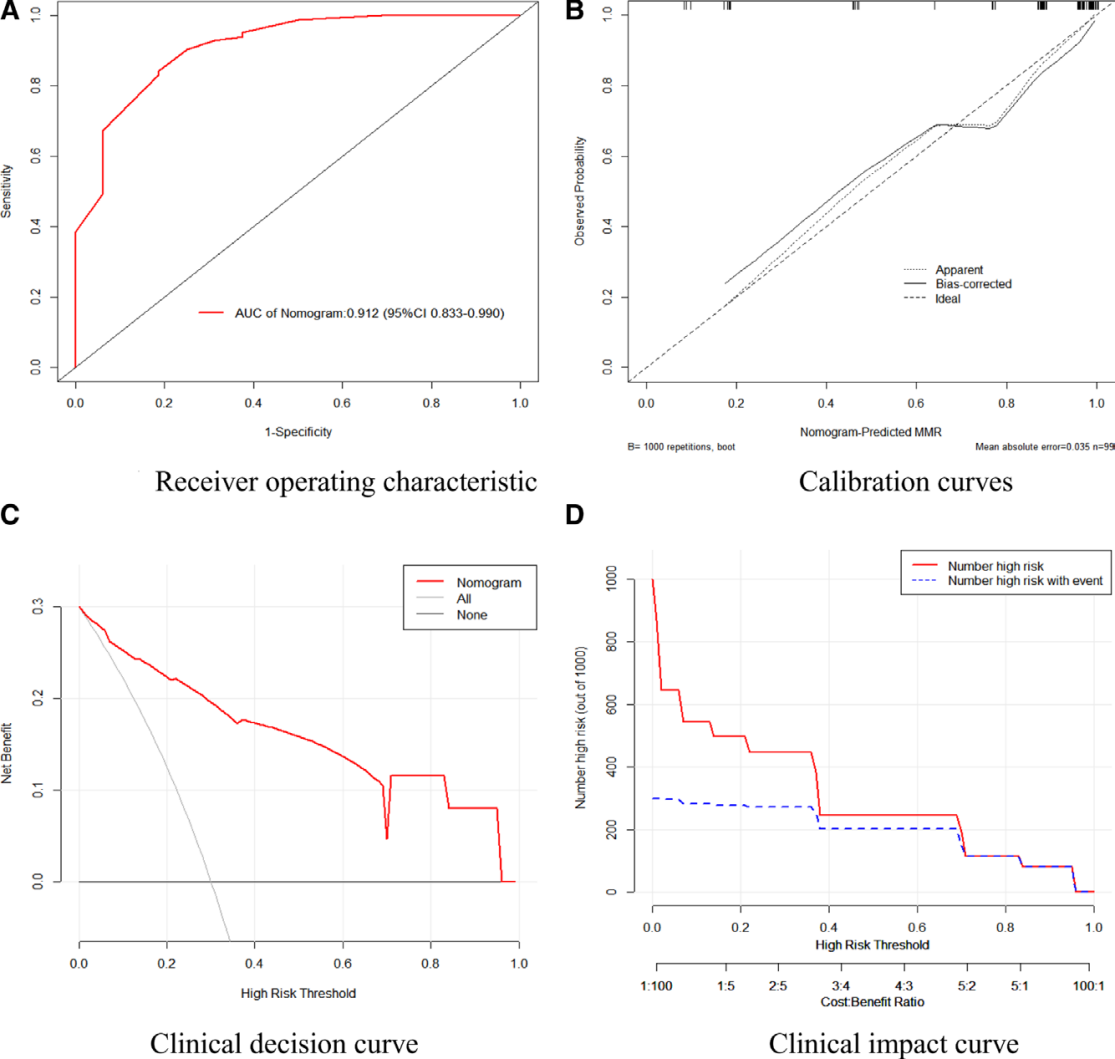
Model fit assessments of MMR. (A) Receiver operating characteristic. (B) Calibration curves. (C) Clinical decision curve. (D) Clinical impact curve. MMR = major molecular response.

The ROC curve (Fig. 3A) analysis for the MMR influencing factor nomogram model exhibits an area under the curve of 0.912 (95% confidence interval: 0.833, 0.990), indicating strong predictive accuracy. The goodness-of-fit test suggests that the model curve closely aligns with the ideal curve, reflecting good calibration and high accuracy (Fig. 3B). The DCA curve (Fig. 3C) shows a higher net benefit for the model compared to 2 reference curves for probabilities between 0.01 and 0.78, supporting its practicality in clinical decision-making within this range. Additionally, the clinical impact curve (Fig. 3D) demonstrates that the model’s predictions closely follow actual occurrences beyond a threshold of 0.7, highlighting high clinical predictive efficiency.

**Figure 4.**
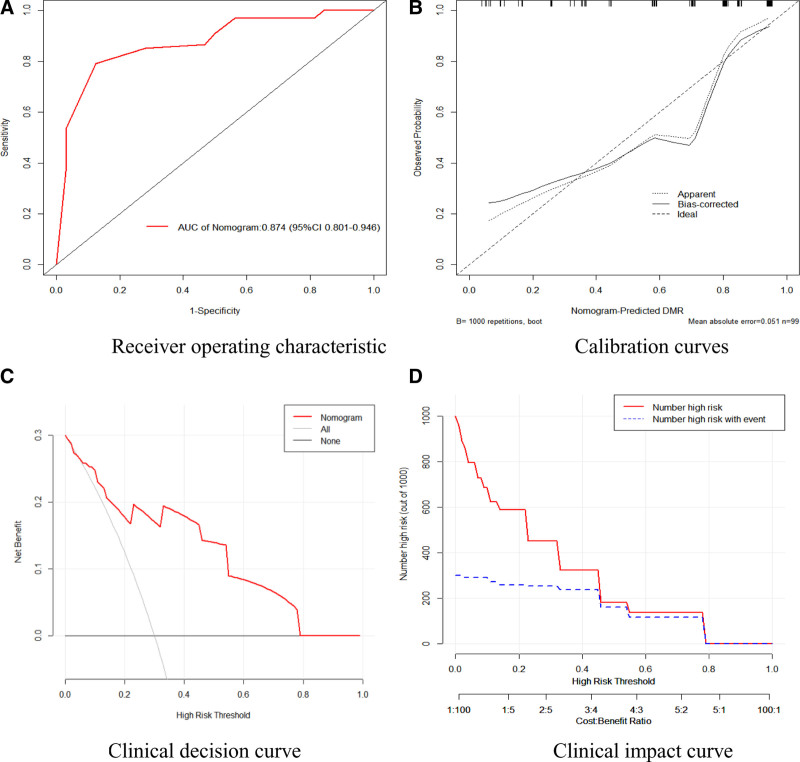
Model fit assessments of DMR. (A) Receiver operating characteristic. (B) Calibration curves. (C) Clinical decision curve. (D) Clinical impact curve. DMR = deep molecular response.

The ROC curve (Fig. 4A) analysis for the DMR influencing factor nomogram model exhibits an area under the curve of 0.874 (95% confidence interval: 0.801, 0.946), indicating strong predictive accuracy. The goodness-of-fit test suggests that the model curve closely aligns with the ideal curve, reflecting good calibration and high accuracy (Fig. 4B). The DCA curve (Fig. 4C) shows a higher net benefit for the model compared to 2 reference curves for probabilities between 0.01 and 0.95, supporting its practicality in clinical decision-making within this range. Additionally, the clinical impact curve (Fig. 4D) demonstrates that the model’s predictions closely follow actual occurrences beyond a threshold of 0.5, highlighting high clinical predictive efficiency.

## 4. Discussion

TKI precisely target the tyrosine kinase encoded by the BCR-ABL fusion gene, effectively blocking the proliferation signaling pathway in CML cells to achieve deep and durable disease control. These mechanisms make TKI highly effective in treating CML.^[8]^

This study analyzed the efficacy of imatinib, nilotinib, and flumatinib in the treatment of CML-CP. At 3 months, CCyR rates for these 3 groups were 53.13%, 76.67%, and 78.38%, respectively, and MMR rates were 25.00%, 53.33%, and 51.35%, respectively. At 12 months, DMR rates were 50.00%, 76.67%, and 75.68%, respectively, marking statistically significant differences (*P* < .05). Compared to imatinib, the CCyR and DMR rates for second-generation TKIs (nilotinib and flumatinib) were higher at multiple monitoring nodes, suggesting that nilotinib and flumatinib may have better long-term therapeutic effects. The second-generation TKIs had a significantly higher MMR rate at 3 months compared to the imatinib group as well. Previous studies^[9]^ have confirmed that early MMR is significantly associated with long-term, progression-free survival. The findings of this study provide strong support for the value of second-generation TKIs in terms of rapid response characteristics, which may improve patients’ long-term prognoses.

While a focus on efficacy is imperative, the occurrence of adverse reactions is also critical issue. In this study, most adverse reactions across the 3 groups were mild to moderate (grades 1–2), with the imatinib group experiencing a higher overall incidence than nilotinib or flumatinib groups. These findings highlight the safety advantages of nilotinib and flumatinib in treating CML.The incidence rates of anemia (31.25%), leukopenia (25.00%), and edema (53.13%) were higher in the imatinib group compared to others; the incidence of abnormal liver function was higher in the nilotinib group (46.67%) and the incidence of diarrhea was higher in the flumatinib group (32.43%). These characteristics of adverse reactions are related to the mechanism of action and metabolic pathways of the drugs.^[10]^ Clinicians must carefully consider the individual conditions of patients when selecting treatment plans, and clinically adjust dosages or switch drugs according to each patient’s tolerance.

Current research suggests that TKI efficacy is influenced by multiple factors. Changes in chromosomal clones, especially mutations in the BCR-ABL kinase domain, directly impact TKI sensitivity.^[11]^ Mutations like T315I are known to confer significant drug resistance. Additionally, studies by Kumar et al^[12]^ underscore the role of the tumor microenvironment in promoting imatinib resistance, as a crucial factor of disease progression, in CML. Accurately identifying both genetic and environmental factors is essential for optimizing CML treatment strategies. Given the high cost of genetic testing, which limits is accessibility, the present study emphasizes a detailed analysis of individual differences at initial diagnoses. By utilizing readily available clinical data to identify influencing factors and construct predictive models, the proposed model may be used to guide personalized CML treatment plans to enhance therapeutic outcomes.

Multi-factor analysis in this study indicated early EMR, WBC, RDW, and PLT as independent influencing factors of MMR. Age, drug type, early EMR, and RDW were identified as independent influencing factors for DMR. The probability of elderly patients (≥60 years old) achieving DMR was significantly reduced (odds ratio [OR] = 0.207) compared to other age groups, which may be related to the decline of immune function, the increase of comorbidities, and differences in drug metabolism occurring with age.^[13]^ Kaiser et al^[14]^ investigated SIRT7 expression and age-related changes in the hematopoietic system to find that aging accelerates the progression of myeloid stem cell diseases (e.g., AML, CML), providing a solid theoretical basis for age as a critical factor in treatment response.

Additionally, Yokoyama et al^[15]^ found a correlation between high initial white blood cell count and increased nm23-H2 mRNA levels in CML patients, suggesting that white blood cell count is an important biomarker of disease progression. Patnaik et al^[16]^ similarly emphasized high white blood cell count as an independent risk factor for poor survival outcomes. PLT at initial diagnosis was found here to be an independent predictor of MMR achievement (OR = 0.115), consistent with the weight of high platelet loads in the Sokal score,^[17]^ suggesting that thrombocytosis may reflect a higher tumor load or a more active disease state.

Furthermore, the results of this study show that early EMR is a powerful predictor of 12-month MMR/DMR (OR = 12.531–45.336). This further supports the ELN guideline^[6,7]^ whereby early EMR is an important node in treatment adjustment. RDW is an emerging predictor, and its potential mechanism may be related to iron metabolism disorder, oxidative stress, and the inflammatory microenvironment.^[18]^

Mao^[19]^ confirmed that elevated RDW level at diagnosis is a predictor of efficacy and poor prognosis independent of other factors. This study found that an increase in RDW was significantly correlated with a decrease in MMR/DMR (OR = 0.152–0.246), consistent with results from previous studies and supporting the clinical predictive value of RDW.

A previous study in the Brazilian population^[20]^ indicated low education level as an important factor preventing CML patients from achieving cytogenetic remission during imatinib therapy. Through single factor analysis, the present study found that education level exerted some influence on therapeutic effects; however, this influence did not remain statistically significant after multivariate analysis. Nonetheless, it is imprudent to rule out the possibility that education level impacts therapeutic efficacy. Theoretically, education may play a role in patients’ adherence to medication, ability to recognize symptoms, and timeliness in seeing a doctor. These factors were not included in the present study due to some limitations in the design and implementation conditions. Therefore, future researchers may consider a more in-depth analysis of these factors.

The MMR/DMR nomogram model constructed in this study demonstrates high prediction and differentiation efficiency, with areas under the ROC curve of 0.912 and 0.874, respectively. The calibration curve confirmed the model’s high predictive accuracy, while decision curve and clinical impact analyses highlighted its precision and substantial clinical decision-making value.

## 5. Summary

This study extensively analyzed the efficacy and safety of TKI in CML treatment, highlighting the importance of clinical characteristics, sociodemographic factors, and adverse drug-related reactions on treatment selection and outcomes. A clinical predictive model based on these factors can be effectively implemented in clinical practice to accurately identify high-risk patient groups, which may facilitate personalized, targeted treatment planning approaches to improve overall therapeutic efficacy for CML patients.

The limitations of this study include a relatively short follow-up period, which did not allow for a comprehensive evaluation of long-term survival differences across groups. Further multicenter prospective cohort studies are yet necessary to extend follow-up timeframes and incorporate more high-risk patients to verify the robustness of the proposed model.

## Author contributions

**Conceptualization:** Xuliang Shen, Zizhong Zhang, Weiwei Jiang.

**Data curation:** Zizhong Zhang, Sen Ding.

**Formal analysis:** Zizhong Zhang, Weiwei Jiang.

**Funding acquisition:** Xuliang Shen.

**Investigation:** Zizhong Zhang, Sen Ding.

**Methodology:** Xuliang Shen, Weiwei Jiang.

**Project administration:** Xuliang Shen.

**Resources:** Xuliang Shen.

**Software:** Zizhong Zhang, Weiwei Jiang.

**Supervision:** Xuliang Shen.

**Writing – review & editing:** Xuliang Shen, Zizhong Zhang, Weiwei Jiang.

**Writing – original draft:** Zizhong Zhang, Weiwei Jiang, Sen Ding.

## References

[1] Qian XL. Prognostic value and efficacy analysis of BCR-ABL and significance of ABL kinase domain mutation in chronic myeloid leukemia after TKI treatment [D]. Soochow University. 2019.

[2] Yayun W Hongguo Z Zhongguang C. 3-year clinical observation of dasatinib, nilotinib and imatinib in the treatment of newly diagnosed chronic myeloid leukemia in chronic phase. Chin J Lab Hematology. 2015;23:356–63.

[3] Cheng YX. Application effect of tyrosine kinase inhibitor in the treatment of chronic myeloid leukemia [D]. Anhui Med Univ. 2016.

[4] Xiaoshuai Z Yaqen Q Yueyun L. Combination of socio-demographic and clinical factors to predict treatment response and outcome in patients with chronic myeloid leukemia in the chronic phase. Chin J Hematol. 2021;43:54–62. 10.3760/cma.j.issn.0253-2727.2022.01.011 PMC8980668 35231994

[5] Arber DA Orazi A Hasserjian R. The 2016 revision to the World Health Organization classification of myeloid neoplasms and acute leukemia. Blood. 2016;127:2391–405. 27069254 10.1182/blood-2016-03-643544

[6] Baccarani M Deininger MW Rosti G. European LeukemiaNet recommendations for the management of chronic myeloid leukemia: 2013. Blood. 2013;122:872–84. 23803709 10.1182/blood-2013-05-501569 PMC4915804

[7] Hochhaus A Baccarani M Silver RT. European LeukemiaNet 2020 recommendations for treating chronic myeloid leukemia. Leukemia. 2020;34:966–84. 32127639 10.1038/s41375-020-0776-2 PMC7214240

[8] Xiong T Hu J. Prognostic factors of chronic myeloid leukemia treated with second-generation TKI. Oncol Pharmacy. 2023;13:332–7.

[9] Saussele S Hehlmann R Fabarius A. Defining therapy goals for major molecular remission in chronic myeloid leukemia: results of the randomized CML Study IV. Leukemia. 2018;32:1222–8. 29479070 10.1038/s41375-018-0055-7 PMC5940636

[10] Massaro F Colafigli G Molica M Breccia M. Novel tyrosine-kinase inhibitors for the treatment of chronic myeloid leukemia: safety and efficacy. Expert Rev Hematol. 2018;11:301–6. 29522367 10.1080/17474086.2018.1451322

[11] Ting Y Yueyun LAI Yaqen Q. Analysis of the efficacy and influencing factors of nilotinib and dasatinib as second- or third-line drugs in the treatment of patients with chronic myeloid leukemia in chronic phase and accelerated phase. Chin J Hematol. 2020;41:93–9. 10.3760/cma.j.issn.0253-2727.2020.02.002 PMC7357953 32135623

[12] Kumar R Pereira RS Zanetti C. Specific, targetable interactions with the microenvironment influence imatinib-resistant chronic myeloid leukemia. Leukemia. 2020;34:2087–101. 32439895 10.1038/s41375-020-0866-1 PMC7387317

[13] Rozental A Halperin E Leibovitch C. CML in the very elderly: the impact of comorbidities and TKI selection in a real-life multicenter study. Ann Hematol. 2024;103:3585–94. 38862792 10.1007/s00277-024-05828-3 PMC11358301

[14] Kaiser A Schmidt M Huber O. SIRT7: an influence factor in healthy aging and the development of age-dependent myeloid stem-cell disorders. Leukemia. 2020;34:2206–16. 32214204 10.1038/s41375-020-0803-3 PMC8318878

[15] Yokoyama A Okabe-Kado J Sakashita A. Differentiation inhibitory factor nm23 as a new prognostic factor in acute monocytic leukemia. Blood. 1996;88:3555–61. 8896423

[16] Patnaik MM Barraco D Lasho TL. Targeted next generation sequencing and identification of risk factors in World Health Organization defined atypical chronic myeloid leukemia. Am J Hematol. 2017;92:542–8. 28314085 10.1002/ajh.24722 PMC5422114

[17] Aijaz J Junaid N Naveed MA. Risk stratification of chronic myeloid leukemia according to different prognostic scores. Cureus. 2020;12:e7342. 32313783 10.7759/cureus.7342 PMC7164706

[18] Ai L Mu S Hu Y. Prognostic role of RDW in hematological malignancies: a systematic review and meta-analysis. Cancer Cell Int. 2018;18:61. 29713244 10.1186/s12935-018-0558-3 PMC5914059

[19] Mao XL Xi YM Li ZJ. Higher red blood cell distribution width at diagnose is a simple negative prognostic factor in chronic phase-chronic myeloid leukemia patients treated with tyrosine kinase inhibitors: a retrospective study. Medicine (Baltim). 2021;100:e24003. 10.1097/MD.0000000000024003 PMC7969257 33725811

[20] Rego MN Metze K Lorand-Metze I. Low educational level but not low income impairs the achievement of cytogenetic remission in chronic myeloid leukemia patients treated with imatinib in Brazil. Clinics (Sao Paulo). 2015;70:322–5. 26039947 10.6061/clinics/2015(05)03 PMC4449460

